# Exploring the therapeutic potential of “Tianyu” medicine pair in rheumatoid arthritis: an integrated study combining LC-MS/MS, bioinformatics, network pharmacology, and experimental validation

**DOI:** 10.3389/fmed.2024.1475239

**Published:** 2024-10-04

**Authors:** Lu Tang, Dingyuan Guo, Dongye Jia, Songlan Piao, Chunqiu Fang, Yueya Zhu, Yinghang Wang, Zhi Pan

**Affiliations:** ^1^Fangzheng Research Laboratory, Jilin Ginseng Academy, Changchun University of Chinese Medicine, Changchun, China; ^2^Department of Traditional Chinese Internal Medicine, Dongzhimen Hospital, Beijing University of Chinese Medicine, Beijing, China; ^3^Department of Pathology Teaching and Research, Clinical Medical School, Changchun University of Chinese Medicine, Changchun, China; ^4^Department of Rheumatology and Immunology, The Affiliated Hospital to Changchun University of Chinese Medicine, Changchun, China

**Keywords:** rheumatoid arthritis, “Tianyu” medicine pair, Chinese medicine, bioinformatics analysis, machine learning

## Abstract

**Background:**

Rheumatoid arthritis (RA) is a widespread chronic autoimmune disease that primarily causes joint inflammation and damage. In advanced stages, RA can result in joint deformities and loss of function, severely impacting patients’ quality of life. The “Tianyu” pair (TYP) is a traditional Chinese medicine formulation developed from clinical experience and has shown some effectiveness in treating RA. However, its role in the complex biological mechanisms underlying RA remains unclear and warrants further investigation.

**Methods:**

We obtained gene sequencing data of synovial tissues from both RA patients and healthy individuals using two gene microarrays, GSE77298 and GSE55235, from the GEO database. Through an integrated approach involving bioinformatics, machine learning, and network pharmacology, we identified the core molecular targets of the “Tianyu” medicine pair (TYP) for RA treatment. Liquid chromatography-mass spectrometry was then employed to analyze the chemical components of TYP. To validate our findings, we conducted animal experiments with Wistar rats, comparing histopathological and key gene expression changes before and after TYP treatment.

**Results:**

Our data analysis suggests that the onset of RA may be associated with inflammation-related immune cells involved in both adaptive and innate immune responses. Potential key targets for TYP treatment in RA include AKR1B10, MMP13, FABP4, NCF1, SPP1, COL1A1, and RASGRP1. Among the components of TYP, Kaempferol, Quercetin, and Salidroside were identified as key, with MMP13 and NCF1 showing the strongest binding affinity to these compounds. Animal experiments confirmed the findings from bioinformatics and network pharmacology, validating the key targets and therapeutic effects of TYP in treating RA.

**Conclusion:**

Our study reveals that TYP has potential clinical value in the treatment of rheumatoid arthritis. This research enhances our understanding of RA’s pathogenesis and provides insight into potential therapeutic mechanisms.

## 1 Introduction

Rheumatoid arthritis (RA) is a systemic, chronic autoimmune disease that primarily affects the joints in the hands and feet, impacting 0.5 to 1.0% of the global population (1). RA usually manifests in individuals between the ages of 50 and 60 and is characterized by symmetric joint pain and swelling, which can lead to joint deformity and progressive damage (2). Traditionally, RA has been viewed as an incurable lifelong condition that requires continuous immune regulation to manage disease progression effectively. Over the past 30 years, the global burden of RA has increased and is projected to rise further. This highlights the importance of prevention and early intervention to mitigate its impact. The progression of RA is unpredictable, marked by phases of symptom exacerbation and remission. Without effective treatment, the disease progressively worsens, resulting in irreversible joint damage and impairments in both physical and psychological health. Moreover, RA is associated with various complications and comorbidities that can reduce life expectancy by several years (3).

Currently, the treatment of rheumatoid arthritis (RA) primarily aims to reduce inflammation, swelling, and pain. Common medications include nonsteroidal anti-inflammatory drugs (NSAIDs), glucocorticoids, and disease-modifying antirheumatic drugs (DMARDs), all of which help manage the disease. However, these treatments lack specificity and often require long-term use, which can result in severe adverse effects. Moreover, the need for injectable medications can reduce patient compliance and increase discomfort (4). Consequently, there is a growing need for new biologics or treatments with minimal side effects, underscoring the importance of ongoing research in this area.

The ‘Tianyu’ drug pair (TYP) consists of *Rhodiola rosea* and *Euonymus alatus*. *Rhodiola rosea* is a traditional Chinese medicine known for its various therapeutic effects, including protection against myocardial ischemia–reperfusion injury, lipid-lowering, and antithrombotic properties (5). It has also been widely utilized in traditional Chinese medicine for treating RA. *Euonymus alatus* (Thunb.) Siebold, another well-known medicinal plant, has been used for thousands of years in China to treat a range of conditions, such as urticaria, dysmenorrhea, wounds, dysentery, blood stasis, rheumatism, and arthritis (6). When incorporated into traditional Chinese medicine formulations, TYP shows notable effectiveness in the clinical treatment of RA.

This paper aims to explore the mechanisms and therapeutic targets of TYP in treating RA through a comprehensive analysis combining bioinformatics, machine learning, network pharmacology, and animal experiments. The study intends to provide both theoretical insights and data to support future RA treatment strategies.

## 2 Materials and methods

### 2.1 TYP drug target acquisition

The active ingredients of the ‘Tianyu’ drug pair (TYP) were identified based on their oral bioavailability (OB) and drug-likeness (DL). For *Euonymus alatus* (also known as ‘Guijianyu’), active ingredients with OB ≥ 30% and DL ≥ 0.18 were selected by searching the Traditional Chinese Medicine Systems Pharmacology Database and Analysis Platform (TCMSP, http://lsp.nwu.edu.cn/tcmsp.php) using ‘*Euonymus alatus*’ as the keyword. The corresponding drug target information was then screened from the database.

For *Rhodiola rosea* (referred to as ‘Hongjingtian’), the effective components were obtained from the High-Throughput Experiment- and Reference-Guided Database of Traditional Chinese Medicine (HERB, http://herb.ac.cn/) using ‘Hongjingtian’ as the keyword. The drug target information for ‘Hongjingtian’ was further identified using Swiss Target Prediction.^1^

### 2.2 Collection of RA target genes

Two gene expression datasets, GSE77298 and GSE55235, were obtained from the GEO database.^2^ The microarray data were standardized for quality and underwent batch effect correction using the “SVA” R package. This preprocessing enabled the identification of target genes associated with RA.

### 2.3 Differentially expressed gene screening

By combining the data from the GSE77298 and GSE55235 microarrays, genes with a *p < 0.05* and |log2FC| > 2 were identified as significantly differentially expressed. Using R software (version 4.3.2), volcano plots and heatmaps were generated to visualize the comprehensive set of differentially expressed genes from the datasets, as well as the specific genes that were carefully selected based on TYP targets.

### 2.4 Enrichment analysis

Based on the targets identified in Section 2.3, we performed enrichment analysis using the “ClusterProfiler” package in R. This included Gene Ontology (GO), Kyoto Encyclopedia of Genes and Genomes (KEGG), and Gene Set Enrichment Analysis (GSEA). Items with *p < 0.05* were considered significant and were subsequently visualized for interpretation (7).

### 2.5 Protein–protein interaction

We constructed a protein–protein interaction (PPI) network using the “Multiple proteins” function in the STRING database^3^, selecting “*Homo sapiens*” as the organism. The network was built based on the overlapping targets identified from the differentially expressed genes screened in TYP and Section 2.3.

### 2.6 Machine learning

We applied Least Absolute Shrinkage and Selection Operator (LASSO) analysis and Random Forest (RF) analysis to the key genes identified in Section 2.3, using the R packages “Glmnet” and “RandomForest,” respectively. The hub genes were determined by identifying the intersection of the results from both analyses.

### 2.7 Immune infiltration analysis

We performed an immune cell infiltration analysis on the differentially expressed genes (DEGs) identified in Section 2.3 using the “ssGSEA” algorithm within the “GSVA” package in R software (version 4.3.2). Comparative box plots and heatmaps were then generated to visualize the immune infiltration patterns across different groups.

### 2.8 Analytical methodology for the detection of TYP using LC–MS/MS

This study presents a methodology for detecting TYP using liquid chromatography–tandem mass spectrometry (LC–MS/MS). Sample preparation involved weighing lyophilized TYP powder into 2 mL centrifuge tubes, adding 600 μL of methanol containing 2-chloro-L-phenylalanine (4 ppm), and vortexing for 30 s. Steel beads were then added, and the mixture was homogenized at 55 Hz for 60 s. Samples were sonicated at room temperature for 15 min, followed by centrifugation at 14,000 x g at 4°C for 10 min. The supernatant was filtered through a 0.22 μm membrane and transferred to vials for LC–MS analysis (8).

Liquid chromatography was conducted on a Vanquish UHPLC system coupled with a Waters ACQUITY UPLC^®^ HSS T3 column maintained at 40°C. The mobile phase consisted of 0.1% formic acid in water and acetonitrile, with a flow rate of 0.3 mL/min. The gradient program started at 8% acetonitrile, increased to 98% over seven minutes, and reverted to the initial conditions by 12 min. Mass spectrometry detection was performed using a Thermo Fisher Q Exactive mass spectrometer in both positive and negative ion modes, with a scanning range of m/z 100–1,000 to ensure precise detection. All reagents were of LC–MS grade, and ultrapure water was obtained from a Milli-Q system to enhance the reliability of the results (9, 10). Subsequently, Gene Ontology (GO) and Kyoto Encyclopedia of Genes and Genomes (KEGG) enrichment analyses were conducted on the detected components.

### 2.9 Molecular docking

The effective components of TYP were initially identified and screened using the TCMSP and HERB databases. These components were then cross-referenced with those identified through LC–MS/MS analysis. Molecular docking was performed between the overlapping components and the target proteins identified via LASSO analysis. The 3D structures of the target proteins were retrieved from the Protein Data Bank (PDB)^4^, while the structures of the small molecule ligands were obtained from the PubChem database.^5^ The docking process was carried out using AutoDock software (version 4.2.6) to calculate binding energies, and the results were visualized using PyMOL software.

### 2.10 Animals

Male Wistar rats (180–200 g) were obtained from Changchun Yisi Experimental Animal Technology Co., Ltd. The experimental procedures involving animals were reviewed and approved by the Animal Experimental Ethics Committee of Changchun University of Chinese Medicine. All methods adhered to the principles of animal protection, welfare, and the 3Rs (Replacement, Reduction, and Refinement) in line with relevant regulations on animal welfare ethics in China. Approval number: 202402.

### 2.11 Preparation of collagen-induced arthritis model and animal administration

Thirty rats were divided into three groups: control, model, and TYP, with 10 rats in each group. Type II bovine collagen was dissolved in 0.1 mol/L acetic acid, filtered through a 0.22 μm filter, and then mixed with pre-cooled Freund’s complete adjuvant in a 1:1 volume ratio. On day 1, a 0.15 mL dose of this mixture was injected into the right hind paw of rats in the model and TYP groups for primary immunization. A booster injection of 0.1 mL of the type II collagen emulsion was administered at the same site on day 7 for secondary immunization.

Throughout the model construction phase, weight changes in the rats were monitored. Based on these changes, RO water was administered daily by gavage to the control and model groups. For the TYP group, a mixture of water and ethanol-extracted TYP powder (crude drug concentration of 8.4 g/kg) was adjusted according to each rat’s weight and administered for 28 days. After 28 days, the rats were anesthetized with ethyl carbamate, and synovial tissue from the right hind paw was collected for experimental analysis.

### 2.12 Western blot analysis

Synovial tissue from the right hind paws of rats in each group was excised and placed into 1.5 mL Eppendorf (EP) tubes. To each tube, 1 mL of RIPA lysis buffer (Beyotime, Shanghai, China) was added, with a ratio of RIPA:PMSF Inhibitor set at 100:1:1. The tissues were finely minced using ophthalmic scissors and lysed for 30 min at 4°C, with vortexing for 25 s every 10 min. Following lysis, the samples were centrifuged at 14,000 x g for 10 min at 4°C, and the supernatant was collected to extract the synovial tissue protein. The protein concentration for each sample was measured using the BCA method (Beyotime, Shanghai, China). Equal amounts of protein were then separated by 10% SDS-PAGE and transferred to polyvinylidene fluoride (PVDF) membranes (Millipore, Bedford, MA). The membranes were blocked with 1% BSA and Tween 20 in TBS at room temperature for 1 h. Subsequently, the membranes were incubated with the primary antibody overnight at 4°C. After incubation, the membranes were washed three times with TBST (TBS with 0.1% Tween 20) for 5–10 min each to remove unbound antibodies, followed by incubation with the HRP-conjugated secondary antibody at room temperature for 1 h. The membranes were washed again in TBST and developed using an enhanced chemiluminescence (ECL) detection kit (Beyotime, Shanghai, China). Images were captured using a chemiluminescence imaging system. *β*-actin was used as an internal control to normalize protein loading, and the intensity of protein bands was quantified using ImageJ software.

### 2.13 Fluorescence quantitative PCR analysis

Human-derived primer sequences were initially located using the Primer Bank database.^6^ The mRNA and protein sequences were then obtained from the NCBI database^7^ to identify the coding DNA sequence (CDS) information for each gene, focusing on the highlighted gene regions. Based on this information, primer sequences were designed using the Primer3 Plus tool.^8^ The designed primer sequences were validated and screened using the BLAST database^9^ to select the primers with the highest relevance. The final selected primers are presented in Table 1.

**Table 1:** Primer sequences used for PCR amplification.

Gene_symbol	Forward sequence	Reverse sequence
Akr1b10	TGTCTGCTACCTTTGAACCG	AGCTTCCTTGACTTTATCTGGG
Mmp13	TCCATCCCGAGACCTCATGT	CTCAAAGTGAACCGCAGCAC
Fabp4	GAATGTGTCATGAAAGGCGTG	AAAACCACCAAATCCCATCAAG
Ncf1	TGTTCCTGGTTAAGTGGCAG	AGGGATGACTCTGTTTTCTGTG
Spp1	AGCCAAGGACCAACTACAAC	CTGAGTGTTTGCTGTAATGCG
Col1a1	GAGACAGGCGAACAAGGTGA	GGGAGACCGTTGAGTCCATC
Rasgrp1	GCCAGCTCCATCTATTCCAAG	ATCCCACAGTCTTTACAGCG

Total RNA was extracted from rat synovial tissues using the RNA Simple Total RNA Kit (Tiangen Biotech Co., Ltd., Beijing, China). The purity and concentration of the isolated RNA were assessed using a NanoPhotometer spectrophotometer (IMPLEN, California, United States). The extracted RNA was then reverse transcribed into complementary DNA (cDNA) according to the manufacturer’s instructions. For the qPCR reaction, a 10 μL mixture was prepared, containing 0.2 μL of each forward and reverse primer, 0.8 μL of target DNA, 5 μL of TB Green Premix Ex Taq (Tli RNaseH Plus) (Takara, Kyoto, Japan), and nuclease-free water to reach a final volume of 10 μL.

### 2.14 Histopathological examination

Following 28 days of continuous intragastric administration of TYP, the body weight of the rats was recorded, and the arthritis index (AI) score was evaluated every 7 days. Photographs of the ankle joints were taken for documentation. After completing the administration period, the spleen, thymus, and leg tissues were harvested to calculate the spleen and thymus indices, defined as follows: Spleen Index = Spleen weight (mg) / Body weight (g), Thymus Index = Thymus weight (mg) / Body weight (g).

**Figure 1:**
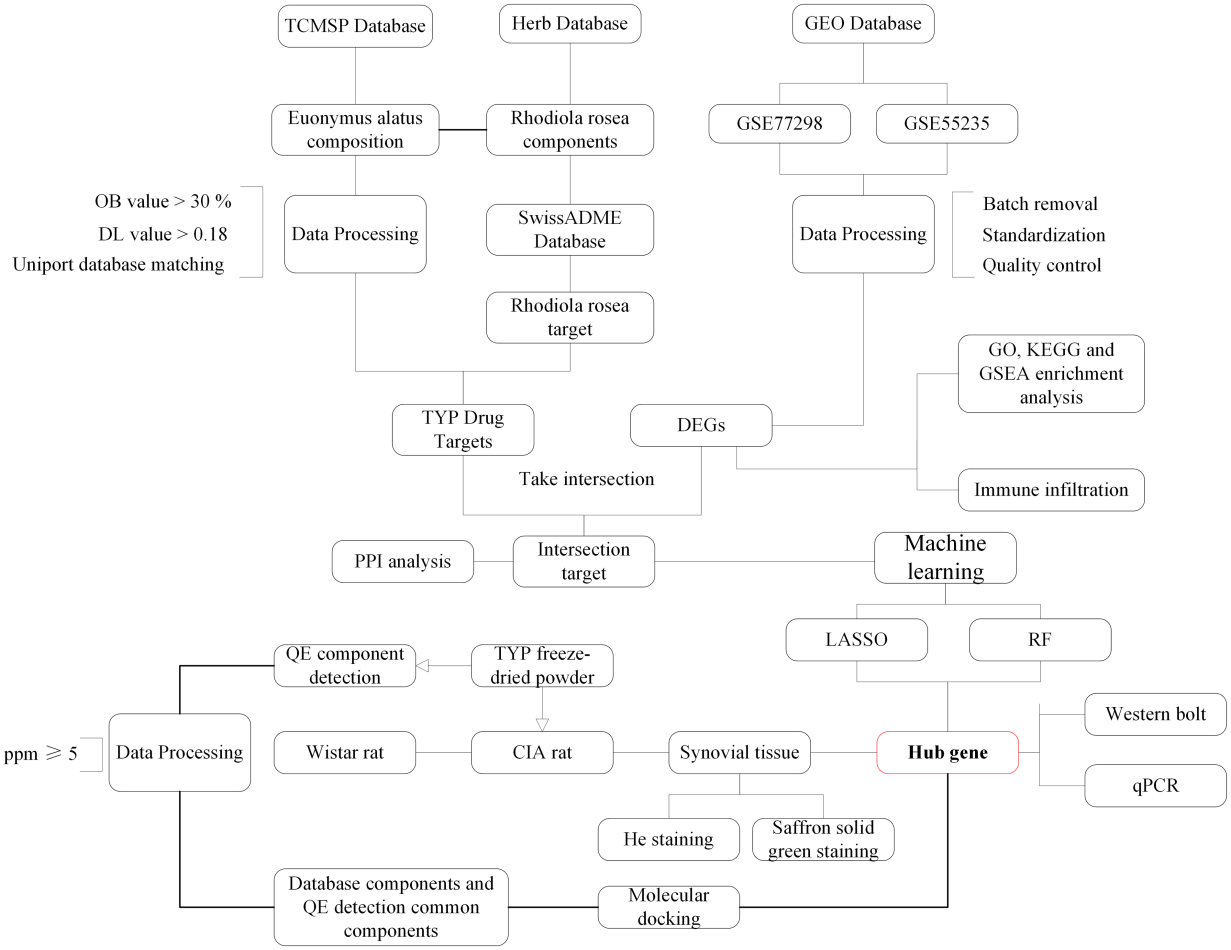
Experimental method flow chart.

Rat knee joints were fixed in 4% paraformaldehyde for 24 h and subsequently demineralized in a 10% ethylenediaminetetraacetic acid (EDTA) solution over two weeks. Following fixation and demineralization, the tissues were dehydrated through a graded series of alcohols, clarified, and embedded in paraffin wax. Consistent 4 μm sections were sliced from these paraffin blocks. The sections were then stained, dehydrated, clarified, and sealed for analysis. Micro-CT scanning was utilized to observe the extent and nature of pathological changes. The tissue sections underwent Hematoxylin and Eosin (H&E) and Safranin O-Fast Green staining to evaluate histopathological features such as joint space architecture, cartilage degradation, and the degree of inflammatory infiltration. The flow chart of the experimental procedure is shown in Figure 1.

## 3 Results

### 3.1 Screening for compounds and targets in TYP

A search in the TCMSP database identified 8 major active ingredients for *Euonymus alatus* (Gujianyu) that met the criteria for oral bioavailability (OB) and drug-likeness (DL). Based on these selected compounds, 177 potential targets were identified from the TCMSP database. For *Rhodiola rosea* (Hongjingtian), a search in the Herb database identified 25 major active ingredients. The SwissTargetPrediction function within the Swiss ADME database was then utilized to predict the targets of these active ingredients, resulting in a total of 624 target entries. After removing duplicates and merging the target data, a comprehensive set of 708 targets associated with the TYP was compiled.

### 3.2 Collection of RA-related genes and overlapping targets

After retrieving and analyzing the data, it was determined that the GSE77298 dataset, based on the GPL570 platform, is titled “Synovial biopsies of rheumatoid arthritis and healthy controls.” This dataset includes 7 control samples and 16 synovial sequencing samples from RA patients. The GSE55235 dataset, using the GPL96 platform, is titled “Identification of rheumatoid arthritis and osteoarthritis patients by transcriptome-based rule set generation.” It contains 10 control samples, 10 osteoarthritis samples, and 10 RA synovial sequencing samples.

**Figure 2:**
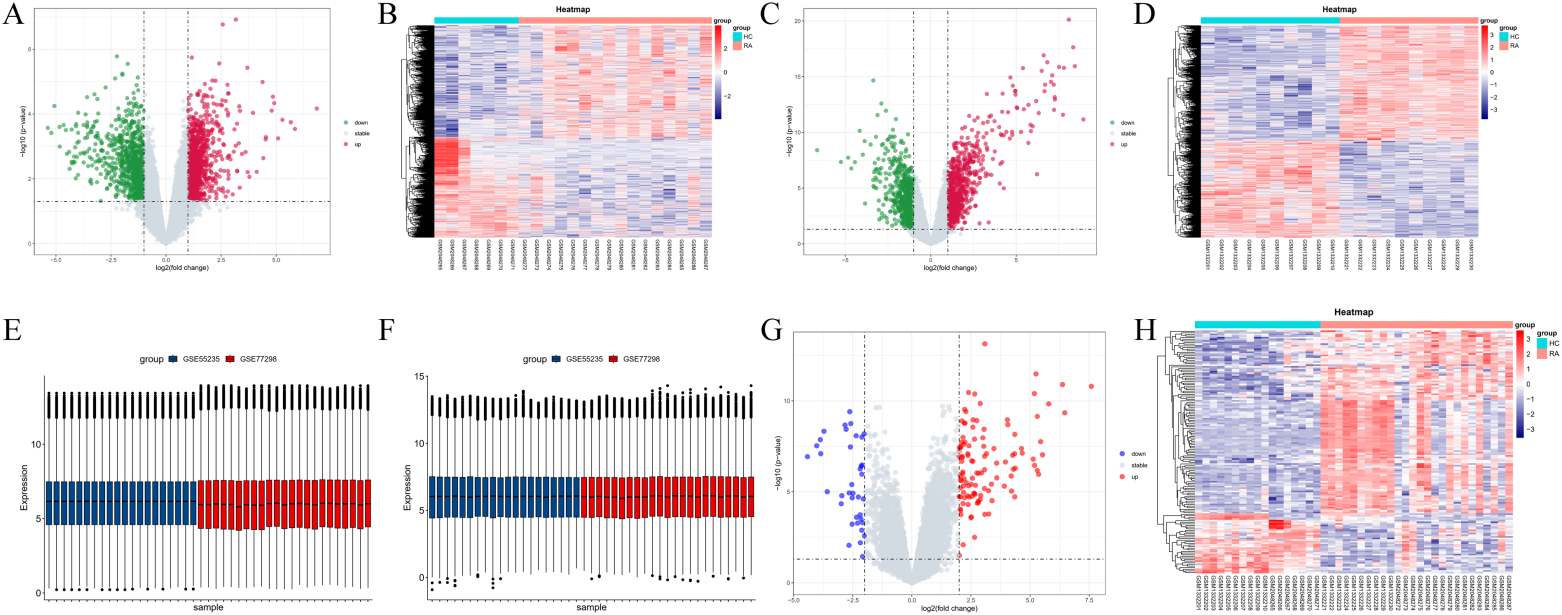
**(A–D)** Volcano plots and heatmaps displaying the differential gene expression between GSE77298 and GSE55235 datasets. **(E,F)** Box plots before and after batch effect removal. **(G,H)** Volcano plots and heatmaps illustrating the differential gene expression after merging the GSE77298 and GSE55235 datasets.

Using R software (version 4.3.2), differential gene volcano plots and heatmaps were generated separately for the GSE77298 and GSE55235 datasets (Figures 2A–D). After correcting for batch effects between the two datasets (Figures 2E,F), the expression levels were normalized, allowing the datasets to be merged into a single comprehensive dataset. From this merged dataset, a total of 150 differentially expressed genes (DEGs) were identified. Among these DEGs, 114 genes were upregulated, and 37 genes were downregulated when comparing the RA group to the normal group, with a significance threshold of LogFC ≥2. Subsequently, combined volcano plots and heatmaps were generated for the integrated data (Figures 2G,H). The DEGs were ranked by the absolute value of LogFC, and the top 10 upregulated and downregulated genes, along with their relevant data, are listed in Tables 2, 3.

**Table 2:** RA up-regulated genes.

	logFC	*p* value	Adj *p* value	Change
IGH	7.590526146	1.58E-11	5.35E-08	Up
IGHG1	6.467247211	4.48E-10	3.19E-07	Up
IGJ	6.368941473	1.26E-11	5.35E-08	Up
IGLC1	5.789130538	1.45E-10	2.18E-07	Up
abParts	5.527537937	9.26E-08	1.24E-05	Up
MMP1	5.444325586	1.74E-08	3.99E-06	Up
IGKV1OR2–108	5.361459216	1.08E-06	7.85E-05	Up
IGLV1-44	5.30699592	7.14E-10	4.59E-07	Up
IGKV1-17	5.304500361	6.62E-07	5.49E-05	Up
CXCL13	5.250714355	3.29E-12	2.22E-08	Up
IGKV1-37	5.188772633	3.50E-07	3.38E-05	Up

**Table 3:** RA down-regulated genes.

	logFC	*p* value	Adj *p* value	Change
PLIN1	−4.416152844	1.16E-07	1.44E-05	Down
CYP4B1	−4.02534239	2.93E-08	5.91E-06	Down
ADH1B	−3.872618138	1.35E-08	3.26E-06	Down
APOD	−3.855799831	7.96E-08	1.13E-05	Down
FABP4	−3.720116264	4.65E-09	1.65E-06	Down
ADIPOQ	−3.580693412	9.80E-06	0.000396	Down
PCK1	−2.989162125	4.52E-05	0.001189	Down
ANGPTL7	−2.964808534	1.62E-05	0.000579	Down
ZBTB16	−2.828062955	2.15E-09	9.36E-07	Down
MYOC	−2.781683207	3.65E-09	1.37E-06	Down

### 3.3 GO, KEGG, and GSEA enrichment analysis

**Figure 3:**
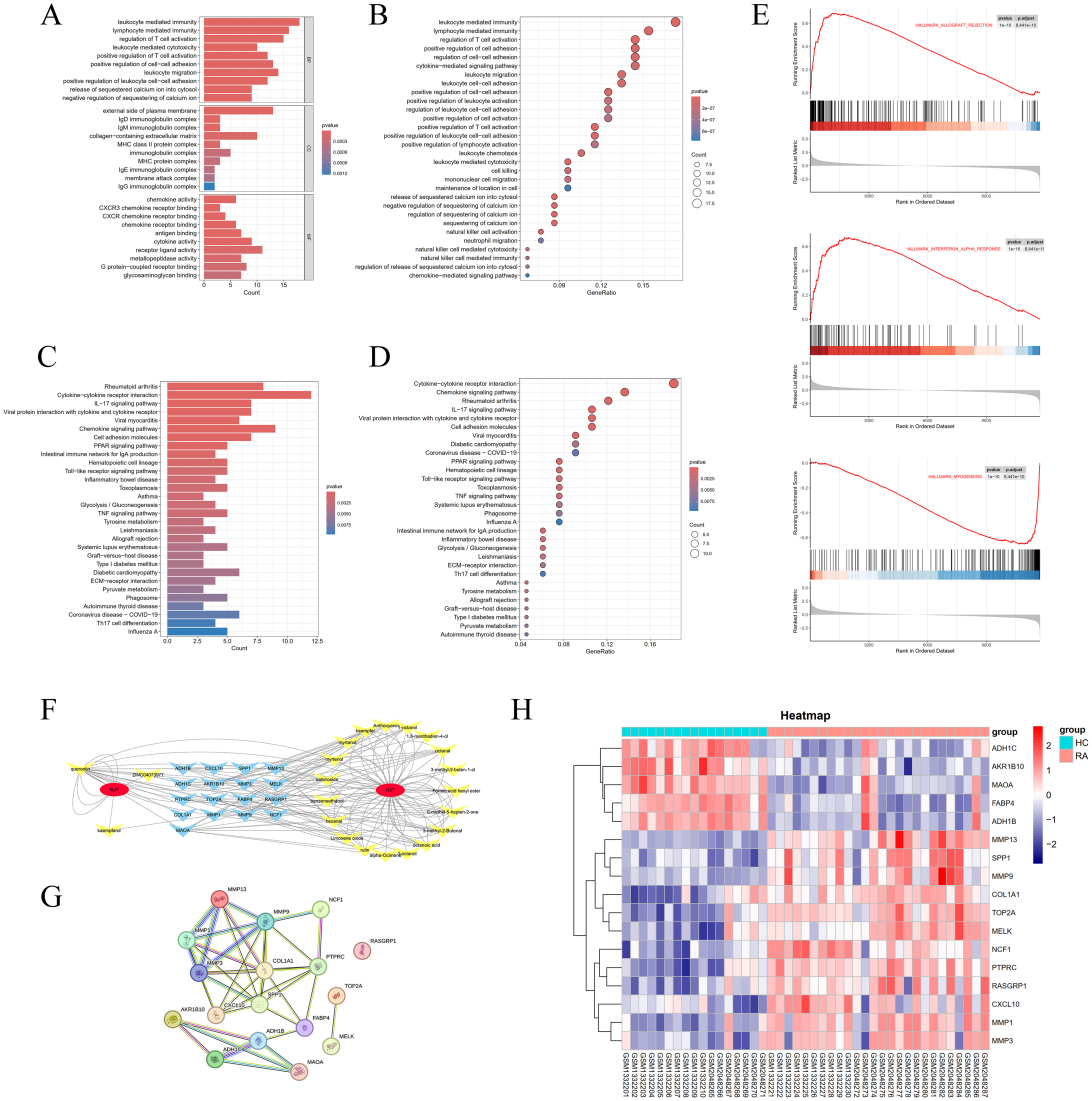
**(A,B)** Bar plots and bubble plots showcasing facetted GO enrichment analysis. **(C,D)** Bar plots and bubble plots depicting KEGG enrichment analysis. **(E)** Curve plot displaying GSEA enrichment analysis. **(F)** Network diagram depicting drug, compound, and target relationships. **(G)** Protein–protein interaction (PPI) network diagram. **(H)** Differential heatmap of duplicate genes in YPY.

The Gene Ontology (GO) enrichment analysis revealed that the differentially expressed genes (DEGs) in RA are primarily enriched in the Biological Process (BP) category, including processes such as leukocyte-mediated immunity, lymphocyte-mediated immunity, and regulation of T cell activation. For Cellular Component (CC) analysis, DEGs are enriched in categories like the external side of the plasma membrane, collagen-containing extracellular matrix, and endocytic vesicle. In the Molecular Function (MF) analysis, DEGs show significant enrichment in receptor-ligand activity, cytokine activity, and G protein-coupled receptor binding (Figures 3A,B).

The Kyoto Encyclopedia of Genes and Genomes (KEGG) pathway analysis indicates that DEGs in RA are significantly enriched in pathways such as cytokine-cytokine receptor interaction, chemokine signaling pathway, IL-17 signaling pathway, and viral protein interaction with cytokines and cytokine receptors (Figures 3C,D).

Moreover, Gene Set Enrichment Analysis (GSEA) performed on the GSE77298 and GSE55235 datasets confirms a strong association of RA with pathways such as Hallmark Allograft Rejection, Hallmark Interferon Alpha Response, and Hallmark Myogenesis (Figure 3E).

### 3.4 Construction of compounds-targets network

By intersecting the genes identified in Section 3.1 with the differentially expressed genes obtained from the analysis in Section 3.2, a total of 17 hub genes were identified (Table 4). After filtering for duplicate genes with active ingredients in TYP, 22 active ingredients were selected (Table 5). The drug-compound-target network was constructed using Cytoscape 3.7.0 software, resulting in a network comprising 41 nodes and 155 edges (Figure 3F). The heatmap analysis of the overlapping genes is shown in Figure 3H.

**Table 4:** Duplicate genes.

Protein name	Gene name
Receptor-type tyrosine-protein phosphatase C	PTPRC
Aldo-keto reductase family 1 member B10	AKR1B10
DNA topoisomerase 2-alpha	TOP2A
Monoamine oxidase type A	MAOA
Matrix metalloproteinase-13	MMP13
Fatty acid-binding protein 4	FABP4
Alcohol dehydrogenase 1B	ADH1B
Matrix metalloproteinase-1	MMP1
Neutrophil cytosol factor 1	NCF1
Sphingosine-1-phosphate phosphatase 1	SPP1
Collagen alpha-1(I) chain	COL1A1
Maternal embryonic leucine zipper kinase	MELK
RAS guanyl-releasing protein 1	RASGRP1
Alcohol dehydrogenase 1C	ADH1C
Matrix metalloproteinase-9	MMP9
Matrix metalloproteinase-3	MMP3
C-X-C motif chemokine 10	CXCL10

**Table 5:** Drugs and their active ingredients.

Drug	Effective components
*Rhodiola Rosea* Components	3-methyl-2-Butenal
*Rhodiola Rosea* Components	6-methyl-5-hepten-2-one
*Rhodiola Rosea* Components	Formic acid hexyl ester
*Rhodiola Rosea* Components	Octanal
*Rhodiola Rosea* Components	1,8-menthadien-4-ol
*Rhodiola Rosea* Components	1-octanol
*Rhodiola Rosea* Components	Anthocyanin
*Rhodiola Rosea* Components	Kaempfer
*Rhodiola Rosea* Components	Myrtanol
*Rhodiola Rosea* Components	Myrtenol
*Rhodiola Rosea* Components	Quercetin
*Rhodiola Rosea* Components	Salidroside
*Rhodiola Rosea* Components	Benzeneethanol
*Rhodiola Rosea* Components	Hexanal
*Rhodiola Rosea* Components	Limonene oxide
*Rhodiola Rosea* Components	Rutin
*Rhodiola Rosea* Components	Alpha-Ocimene
*Rhodiola Rosea* Components	3-octanol
*Rhodiola Rosea* Components	Octanoic acid
*Rhodiola Rosea* Components	3-methyl-2-buten-1-ol
*Euonymus Alatus* Composition	Quercetin
*Euonymus Alatus* Composition	Kaempferol
*Euonymus Alatus* Composition	ZINC04073977

### 3.5 Construction of PPI network

The 17 hub genes identified in Section 3.4 were imported into the STRING database to construct a protein–protein interaction (PPI) network (Figure 3G). This network provides a basis for further exploration of protein interactions related to the targets. Detailed information regarding the PPI network can be found in Table 5.

### 3.6 Identification of candidate hub genes via machine learning

**Figure 4:**
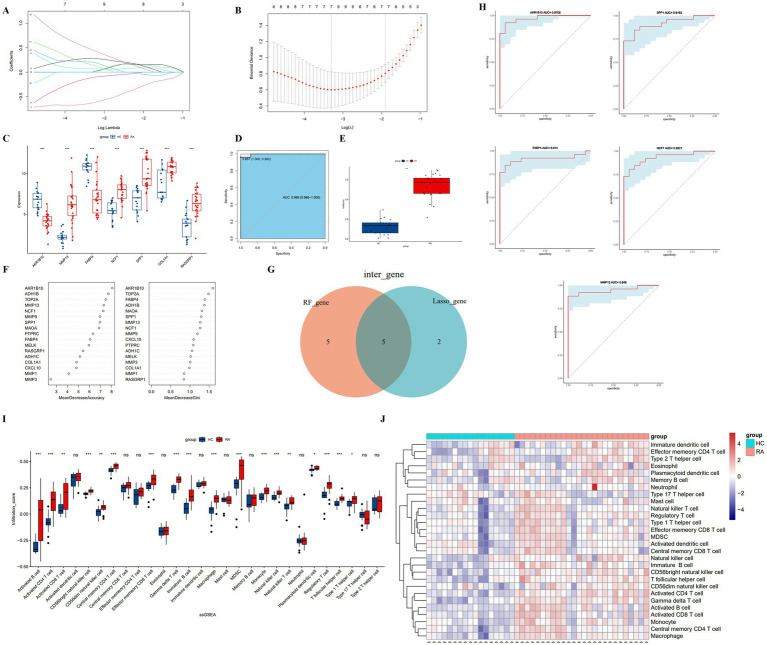
**(A)** Shrinkage plot of regression coefficients with penalty term. **(B)** Optimal *λ* value. **(C)** Box plot of differential gene expression using Lasso. **(D)** ROC curve of risk score. **(E)** Box plot of differential risk scores. **(F)** Gene importance ranking using random forest. **(G)** Intersection of top 10 genes from random forest and Lasso. **(H)** The ROC curve of important genes. **(I,J)** Box plots and heatmaps comparing immune infiltration groups using ssGSEA.

Using the Least Absolute Shrinkage and Selection Operator (LASSO) algorithm, we generated a coefficient profile plot (Figure 4A) and determined the optimal *λ* value (Figure 4B). From these analyses, we identified seven features with non-zero coefficients: AKR1B10, MMP13, FABP4, NCF1, SPP1, COL1A1, and RASGRP1 (Figure 4C). The Area Under the Curve (AUC) and the 95% confidence interval (CI) for these genes were calculated in the LASSO regression analysis, with the resulting Receiver Operating Characteristic (ROC) curve showing an AUC of 0.995 (95% CI: 0.985–1.000) (Figures 4D,E).

For the Random Forest (RF) algorithm, we evaluated the importance of hub genes using metrics such as Mean Decrease Accuracy (MDA) and Mean Decrease Gini (MDG) (Figure 4F). By intersecting the results from both the LASSO and RF analyses, we identified five key genes: AKR1B10, FABP4, MMP13, SPP1, and NCF1 (Figure 4G).

### 3.7 Diagnosis value evaluation

The diagnostic performance of the identified genes for RA was further validated using the GSE55235 and GSE77298 datasets. Receiver Operating Characteristic (ROC) curves were constructed, and the Area Under the Curve (AUC) along with the 95% confidence interval (CI) were calculated for each gene, as shown in Figure 4H. The results demonstrated high diagnostic value for all genes: AKR1B10 (AUC = 0.9706), FABP4 (AUC = 0.914), SPP1 (AUC = 0.9163), MMP13 (AUC = 0.948), and NCF1 (AUC = 0.9321).

### 3.8 Immune infiltration analysis

We utilized the single-sample Gene Set Enrichment Analysis (ssGSEA) algorithm to evaluate the infiltration levels of 28 types of immune cells. The analysis indicated that 18 types of immune cells are potentially involved in the pathogenesis of rheumatoid arthritis. The box plot illustrating immune cell infiltration levels is shown in Figure 4I, and the corresponding heatmap is displayed in Figure 4J.

### 3.9 Qualitative analysis results of TYP drug components by quadrupole mass spectrometry

**Figure 5:**
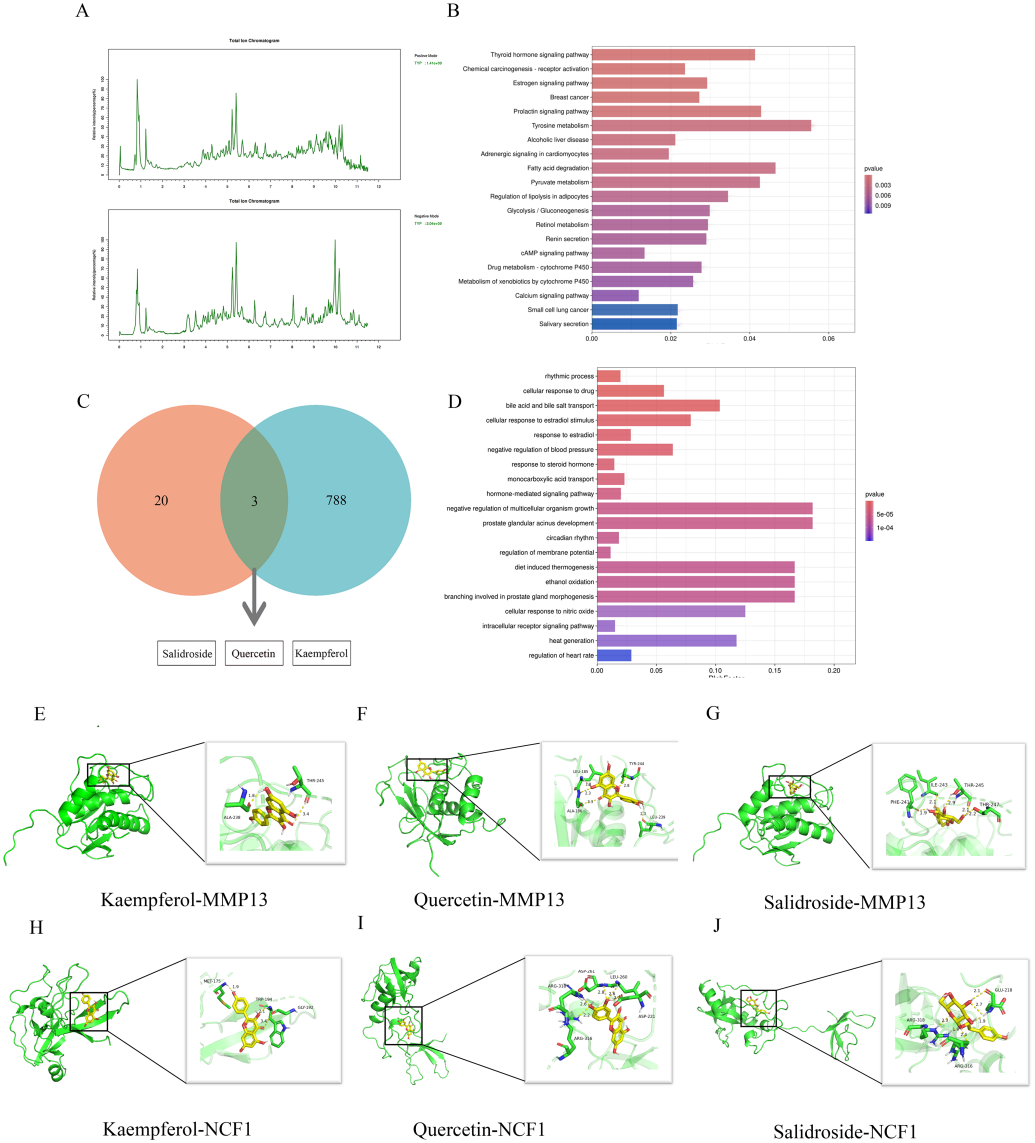
LCMS analysis and molecular docking study of TYP. **(A)** Positive ion flow chart and negative ion flow chart. **(B)** KEGG enrichment analysis of LCMS analysis results of TYP. **(C)** The intersection of LCMS analysis results of TYP and the possible active ingredients of TYP in the treatment of RA in the database. **(D)** GO enrichment analysis diagram of LCMS analysis results of TYP. **(E–J)** 6 groups of specific molecular docking results.

Using the method outlined in Section 2.8, we conducted a compositional analysis of TYP lyophilized powder, identifying a total of 143 components. The total ion chromatogram is presented in Figure 5, with detailed data provided in the Supplementary Table S1. Specifically, Figure 5A displays the positive ion chromatogram, and Figure 5B shows the negative ion chromatogram. The KEGG and GO enrichment analysis bar graphs are illustrated in Figures 5C,D. The intersection of LC–MS analysis results with the effective components screened from the database is presented in Figure 5E.

### 3.10 Molecular docking validation

Three active components of TYP—Kaempferol, Quercetin, and Salidroside—identified in Section 3.9 were selected for molecular docking with five core targets: AKR1B10, FABP4, MMP13, SPP1, and NCF1. The binding energy data are presented in Table 6. A binding energy of ≤5.0 kcal/mol indicates relatively good binding activity, while a binding energy of ≤7.0 kcal/mol suggests strong binding activity (11). In our results, the binding energies of all component-target pairs were > 5.0 kcal/mol, and the MMP13-related binding energies were all ≤7.0 kcal/mol. The strongest binding interaction was observed between MMP13 and NCF1. Specific docking results are shown in Figures 5E–J.

**Table 6:** PPI analysis results.

Gene Name	Average shortest path length	Betweenness centrality	Closeness centrality	Degree
COL1A1	1.615385	0.136538	0.619048	8
SPP1	1.615385	0.136538	0.619048	8
MMP9	1.923077	0.059615	0.52	8
PTPRC	1.692308	0.153846	0.590909	7
MMP3	2	0.008333	0.5	7
CXCL10	2.076923	0.002564	0.481481	6
MMP1	2.076923	0.002564	0.481481	6
MMP13	2.153846	0	0.464286	5
ADH1B	2.153846	0.384615	0.464286	4
FABP4	1.692308	0.461538	0.590909	4
AKR1B10	2.923077	0	0.342105	3
MAOA	2.923077	0	0.342105	3
ADH1C	2.923077	0	0.342105	3
NCF1	2.384615	0	0.419355	2
MELK	1	0	1	1
TOP2A	1	0	1	1

### 3.11 Quantitative PCR analysis of AKR1B10, MMP13, FABP4, NCF1, SPP1, COL1A1, and RASGRP1 expression in synovial tissues of TYP-treated collagen-induced arthritis rats

**Figure 6:**
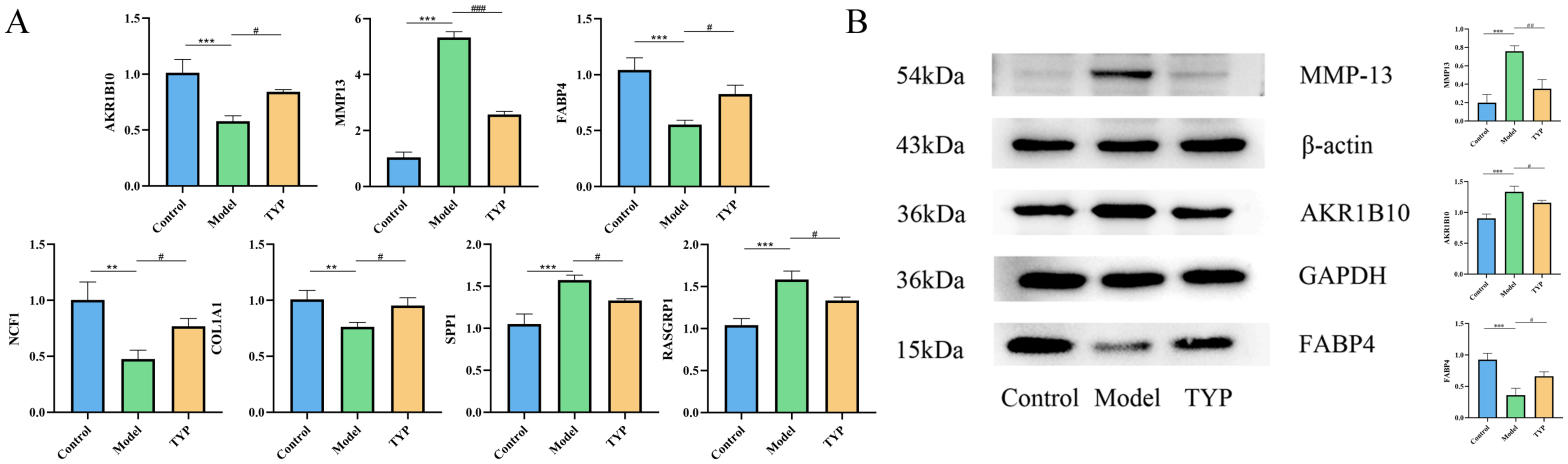
Changes of relevant indexes in synovial tissue of CIA rats after TYP administration. **(A)** The mRNA relative expression levels of AKR1B10, MMP13, FABP4, NCF1, SPP1, COL1A1, and RASGRP1 in the synovium of CIA rats after TYP administration were detected by qPCR (*n* = 3). **(B)** Changes in the protein levels of AKR1B10, MMP13, and FABP4 in the synovial tissue of CIA rats after TYP administration, as determined by Western blotting (*n* = 3). The symbols indicate statistical significance: *p < 0.05, **p < 0.01, compared to the control group; # *p* < 0.05, ## *p* < 0.01 compared to the model group.

Quantitative PCR analysis was performed to evaluate the mRNA expression levels of AKR1B10, MMP13, FABP4, NCF1, SPP1, COL1A1, and RASGRP1 in synovial tissues from collagen-induced arthritis (CIA) rats treated with TYP. Compared to the control group, the model group showed a significant decrease in the relative mRNA expression levels of AKR1B10, FABP4, NCF1, and COL1A1, while the expression levels of MMP13, SPP1, and RASGRP1 were significantly increased (*p < 0.05*). In contrast, the TYP-treated group exhibited a significant restoration of the mRNA expression levels of all the examined genes compared to the model group (*p < 0.05*), as shown in Figure 6A.

### 3.12 Effect of TYP on the protein expression of AKR1B10, FABP4, and MMP13 in synovial tissues of collagen-induced arthritis rat model

Based on the targets identified through LASSO and Random Forest analyses, and ranked by Mean Decrease Gini values, the top three genes (AKR1B10, MMP13, FABP4) were selected for validation through Western blot experiments. The Western blot results demonstrated that, compared to the control group, the expression levels of AKR1B10 and MMP13 were significantly increased in the model group (*p < 0.05*), while the expression level of FABP4 was significantly decreased. Treatment with TYP reversed these changes (*p < 0.05*), as shown in Figure 6B. These findings suggest that TYP has a mitigating effect on the expression changes of AKR1B10, FABP4, and MMP13 in CIA rats (see Table 7).

**Table 7:** Molecular docking information of core ingredients and core targets.

NO.	Protein	PDB ID	Structure	Docking compound	Binding energy (kcal/mol)
1	AKR1B10	1zua	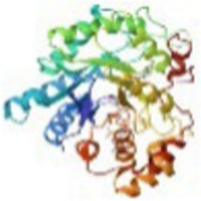	Kaempferol	−7.5
				Quercetin	−6.7
				Salidroside	−7.4
2	FABP4	2hnx	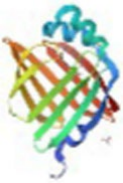	Kaempferol	−7.8
				Quercetin	−6.3
				Salidroside	−7.2
3	MMP13	1xuc	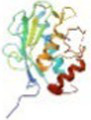	Kaempferol	−10.1
				Quercetin	−10.4
				Salidroside	−8.5
4	SPP1	α-fold	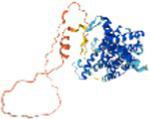	Kaempferol	−7.5
				Quercetin	−7.1
				Salidroside	−7
5	NCF1	1kq6	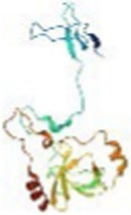	Kaempferol	−7.9
				Quercetin	−7.5
				Salidroside	−6.9

### 3.13 Assessment of TYP effects on swelling, spleen and thymus indices, and histopathological changes in CIA rats

**Figure 7:**
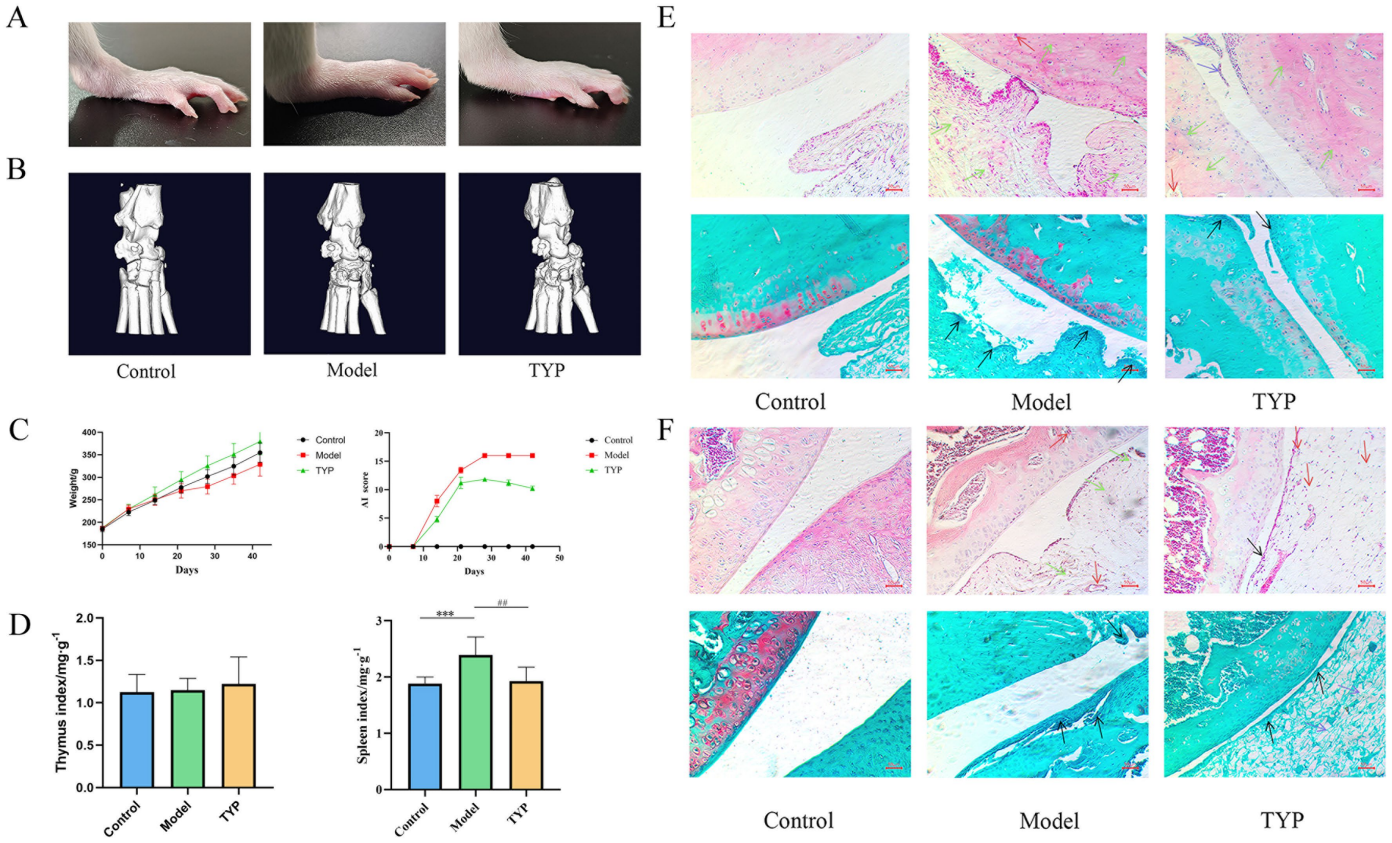
Histopathological examination results: **(A)** Rat ankle joint photography; **(B)** Rat ankle joint microCT imaging results; **(C)** Changes in rat body weight and AI score during TYP gavage; **(D)** Immune organ indices of rat thymus and spleen; **(E)** HE staining and Safranin O/Fast Green staining results of rat ankle joints; **(F)** HE staining and Safranin O/Fast Green staining results of rat knee joints. Black → represents synovial villi, → represents inflammatory infiltration, → represents pannus and tissue congestion, → represents synovial cells. The symbols indicate statistical significance: * *p < 0.05*, ** *p < 0.01*, compared to the control group; # *p < 0.05*, ## *p < 0.01* compared to the model group.

Photographic analysis of rat ankle joints showed severe swelling in the model group compared to the blank group, while the TYP-treated group exhibited reduced swelling relative to the model group (Figure 7A). The arthritis index (AI) score and body weight statistics revealed that the AI score in the TYP group showed a decreasing trend after two weeks of treatment compared to the model group, though there was no significant difference in body weight growth rates among the three groups. The spleen index was significantly higher in both the model and TYP groups compared to the blank group (*p < 0.05*), while there were no statistically significant differences in the thymus index among the groups (Figures 7C,D).

Micro-CT results demonstrated severe bone and cartilage tissue damage in the model group compared to the blank group, characterized by rough bone surfaces and significant cartilage damage. In contrast, the TYP group showed improvements in these symptoms compared to the model group (Figure 7B).

Histopathological examination using Hematoxylin and Eosin (HE) staining showed that, compared to the blank group, the knee and ankle joints of the model group exhibited synovial hyperplasia, inflammatory cell infiltration, proliferation of synovial surface cells and villi, disrupted cartilage structure, and increased lymphocyte infiltration in the subchondral layer. Additionally, there was marked vasodilation and congestion in the interstitium, with some pannus formation in the subchondral layer of the synovium. Safranin O/Fast Green staining in the model group revealed lighter safranin O staining, significant joint swelling, abnormal cartilage structure, disordered arrangement, and proliferation of synovial surface cells and villi. These findings were consistent with previous experimental results, further demonstrating that TYP can alleviate histopathological changes in the CIA rat model (12).

Compared to the model group, the TYP group showed a reduction in synovial surface cell proliferation and villi, slightly abnormal but less disorganized cartilage structure, reduced lymphocyte infiltration in the subchondral layer, and decreased vasodilation and congestion in the interstitium, with no visible pannus in the subchondral layer of the synovium. Safranin O/Fast Green staining in the TYP group indicated slight reductions in joint swelling, mildly abnormal cartilage structure, and reduced proliferation of synovial surface cells and villi (Figures 7E,F).

## 4 Discussion

RA is a chronic, progressive inflammatory disease characterized by symmetric polyarthritis that affects both small and large joints. As a systemic autoimmune disorder, RA has a profound impact on patients’ quality of life by causing structural damage to the joints and surrounding tissues and contributing to systemic inflammation (13). The current pharmacological treatment of RA follows three main principles: the use of nonsteroidal anti-inflammatory drugs (NSAIDs), disease-modifying antirheumatic drugs (DMARDs) to slow disease progression, and glucocorticoids for symptomatic relief (14). However, these treatments are often associated with significant side effects, poor patient compliance, and the need for long-term or lifelong use, resulting in substantial economic and psychological burdens for patients. Consequently, improving early diagnostic methods and developing safer medications with fewer side effects have become primary objectives in RA research. In this study, we utilized bioinformatics analysis and machine learning techniques to evaluate the diagnostic and therapeutic potential of the Tianyu Pair (TYP) in RA patients. Through this approach, we identified three key components of TYP—Kaempferol, Quercetin, and Salidroside—and five crucial immune-related candidate genes: AKR1B10, MMP13, FABP4, NCF1, and SPP1.

Research indicates that Kaempferol, Quercetin, and Salidroside can inhibit the proliferation and migration of rheumatoid arthritis fibroblast-like synoviocytes (RA-FLSs) and mitigate inflammation and synovial damage in RA rat models through various pathways, including PI3K/AKT, MMPs, and NF-κB (15–17). Furthermore, our study has identified several effective compounds that were not previously highlighted in existing databases, such as Curcumin, Cistanche acid, and Tauroursodeoxycholic acid. Curcumin has been shown to induce macrophage apoptosis and reduce inflammatory responses in RA rat models by modulating the NF-κB and mTOR pathways (18, 19). Cistanche acid may inhibit the proliferation of RA-FLS cells and promote apoptosis by activating the Nrf2/HO-1 signaling pathway (20). Tauroursodeoxycholic acid can control RA directly or indirectly—via metabolites like Tau-chloramine, Tau-bromamine, taurine-conjugated cholic acid, and taurine-conjugated raloxifene—through mechanisms such as reducing inflammation, inhibiting oxidative stress, and inducing apoptosis. Additionally, studies have demonstrated that Tauroursodeoxycholic acid exerts anti-apoptotic effects on chondrocytes derived from osteoarthritis (OA) patients when stimulated by H2O2 (21).

In addition to the effective components of TYP, understanding the roles of each key gene in RA treatment provides a more direct and precise perspective. AKR1B10, a member of the aldo-keto reductase (AKR) 1B subfamily, is a human nicotinamide adenine dinucleotide phosphate (NADPH)-dependent reductase. It is involved in the reduction of aldehydes, certain ketones, and quinones. The regulation of the AKR1B10 gene is closely associated with the transcription factor Nrf2. The 5′-flanking region of the AKR1B10 gene contains at least five potential antioxidant response elements (AREs) that respond to Nrf2. Notably, the ARE-A sequence, located between −530 and − 520 bp, exhibits synergistic effects with the adjacent AP-1 site. Due to the resemblance between the AP-1 site and ARE, the tandem arrangement of these two elements enhances the gene’s responsiveness to Nrf2, playing a crucial role in AKR1B10 regulation under various Nrf2-mediated stimuli (22). Previous studies have established a strong link between oxidative stress and chronic inflammation (23), with Nrf2 acting as a central regulator of antioxidative responses. Research has shown a significant increase in Nrf2 expression when RA patients are exposed to oxidative stress (24). Reactive oxygen species (ROS) generated during oxidative stress can contribute to synovial inflammation and bone resorption, promoting the progression of RA (25). Experimental findings from Liu et al. (26) have demonstrated that the AKR1B10 gene contains several regulatory motifs for NF-κB. Moreover, AKR1B10 induces the production of inflammatory cytokines IL1α and IL6 by activating the NF-κB signaling pathway. These inflammatory pathways, particularly those involving the IL-1 family and IL-6, have been shown to be key contributors to the development and progression of rheumatoid arthritis (27–29).

Matrix metalloproteinase 13 (MMP-13) is a key enzyme involved in the degradation of cartilage and bone, making it a crucial protease in the pathogenesis of RA. Patients with RA exhibit significantly elevated levels of MMP-13 expression in their synovial membranes (30). The study by Boldeanu et al. demonstrated a direct correlation between high MMP-13 expression levels and increased disease activity scores in RA (31). Research by Kim et al. suggests that elevated MMP-13 levels in the synovial fluid of RA patients may be linked to articular cartilage degradation and synovial cell proliferation during the progression of the disease. Moreover, their study found that MMP-13 levels in RA synovial fluid are significantly higher than those in patients with osteoarthritis (OA), making MMP-13 a useful marker for distinguishing between RA and OA in diagnostic evaluations (32). Unlike other MMPs, MMP-13 is predominantly expressed in connective tissues. Qin et al. observed that inhibiting MMP-13 production reduces fibroblast-like synoviocyte (FLS) activation and inflammatory responses, underscoring its potential as a therapeutic target (33). The OPG/RANKL ratio represents the balance between osteoprotegerin (OPG), an inhibitor of bone resorption, and the receptor activator of nuclear factor κB ligand (RANKL), a promoter of bone resorption, in bone tissue. A decrease in the OPG/RANKL ratio indicates either reduced OPG or increased RANKL levels, potentially leading to enhanced bone resorption, chondrocyte apoptosis, and matrix loss. The heightened activity of MMP-13, which degrades the extracellular matrix of cartilage, further compromises joint function. MMP-13 activity is also influenced by factors such as abnormal mechanical loading, a pro-inflammatory environment, and a reduced OPG/RANKL ratio. Thus, a decrease in the OPG/RANKL ratio may contribute to upregulated MMP-13 activity, accelerating cartilage degradation and disease progression. Therefore, maintaining an appropriate OPG/RANKL ratio and controlling MMP-13 activity are crucial for preserving cartilage health and preventing the development of bone and joint diseases (34).

Fatty acid-binding protein 4 (FABP4) is a novel adipokine known for its role in regulating inflammation and angiogenesis. It has been extensively studied across various organs and diseases, with numerous studies highlighting the interplay between adipokines, inflammation, and angiogenesis (35). FABP4 has been found to be upregulated in patients with RA, and there is a positive correlation between the expression levels of FABP4 and the severity of the disease (36, 37). According to research by Guo et al. (38), FABP4 is upregulated in the synovial tissue, cartilage, and serum of both RA patients and mouse models. As a major effector cytokine secreted by M1-polarized macrophages, FABP4 promotes macrophage proliferation and M1 polarization. It also enhances the formation of Human Umbilical Vein Endothelial Cell (HUVEC) tubes, facilitates the proliferation, migration, and invasion of HUVECs and fibroblast-like synoviocytes (FLS), and stimulates the release of inflammatory cytokines, thereby disrupting chondrocyte homeostasis. The increased angiogenesis mediated by FABP4 is linked to the exacerbation of RA progression.

Neutrophil Cytosolic Factor 1 (NCF1) encodes a subunit of the NADPH oxidase complex and plays a crucial role in the immune system. A single-nucleotide polymorphism (SNP) in NCF1 has been identified as a modulator of arthritis severity, establishing NCF1 as a genetic factor associated with RA, as observed in rodent models of arthritis. NCF1 encodes the p47phox subunit of the phagocyte NADPH oxidase (NOX2) complex, which is responsible for producing reactive oxygen species (ROS) in immune cells, including antigen-presenting cells. The arthritis-modulating effect of the SNP in NCF1 is linked to an altered capacity of the NOX2 complex to generate ROS (39, 40). A study by Olsson et al. (41) suggested that an elevated copy number of the NCF1 gene may provide a protective effect against the development of RA.

Secreted Phosphoprotein 1 (SPP1), also known as osteopontin, is a multifunctional secreted phosphorylated glycoprotein involved in various cellular processes (42). In collagen-induced arthritis, fibroblast-like synoviocytes (FLS) secrete SPP1, which can activate the PI3K/AKT signaling pathway. This activation promotes the formation and activation of osteoclasts, leading to joint bone destruction, a significant factor in the pathogenesis of arthritis (43, 44). In the synovium of RA patients, SPP1-positive macrophages are present and enriched with cytoskeletal proteins and integrins, highlighting their migratory properties. Upon cleavage, SPP1 exposes epitopes for integrin receptors, and in calcium-activated macrophages, thrombin expression is upregulated. The concentration of cleaved SPP1 is higher in the supernatant of calcium-activated macrophages from RA patients. Furthermore, SPP1 strongly induces the pro-inflammatory activation of monocytes, thereby exacerbating the inflammatory response (45).

Collagen type I alpha 1 (COL1A1) encodes the primary subunit of type I collagen, the predominant structural protein in vertebrates. Numerous mutations in COL1A1 are associated with conditions such as osteoporosis and osteogenesis imperfecta (46, 47). Although COL1A1 is well-studied in relation to these diseases, its role in rheumatoid arthritis (RA) has not been extensively explored. There are limited studies investigating the therapeutic potential of targeting COL1A1 in RA, and the underlying mechanisms remain to be fully elucidated.

Ras Guanyl-Releasing Protein 3 (RasGRP3) is currently regarded as a biomarker associated with the response to TNF-*α* inhibitors. In patients with RA, RasGRP3 expression levels are observed to increase under the influence of TNF-α, suggesting that RasGRP3 can be modulated by TNF-α in B cells (48). Research has also demonstrated a direct correlation between Ras Guanine Nucleotide-Releasing Factor 1 (RasGRF1) and the production of matrix metalloproteinases (MMP-1 and MMP-3), indicating a significant role for RasGRF1 in promoting inflammation and joint degradation in RA. By manipulating RasGRF1 expression through upregulation and silencing, it has been found that RasGRF1 directly contributes to MMP-3 production. Additionally, regulating RasGRF1 expression could significantly impact the semi-transformed phenotype of RA synovial fibroblasts (49).

According to immune infiltration results, the incidence of rheumatoid arthritis (RA) is primarily associated with inflammation-related immune cells involved in both adaptive and innate immune responses. In autoimmune diseases, B cells are generally considered to be key drivers of pathogenesis, contributing to the initiation and persistence of immune responses that lead to chronic synovitis (50). Research highlights the crucial role of B lymphocytes in RA pathogenesis; they produce rheumatoid factors and anti-citrullinated protein antibodies, which contribute to the formation of immune complexes and complement activation within the joints. B cells also act as antigen-presenting cells, producing factors and antibodies that promote inflammation and joint damage (51). Additionally, B cells are a source of autoantibodies that contribute to the formation of immune complexes and complement activation in the joints, as well as the activation of T cells. The immunogenetics of RA suggest that abnormal T-cell activation pathways play a crucial role in the initiation and perpetuation of the disease. During T-cell activation, CD4+ T cells interact with antigenic peptide fragments presented in complex with HLA class II molecules, along with co-stimulatory molecules such as CD80/CD86, which are expressed on the surface of professional antigen-presenting cells (52). T cells are key components of the immune cell infiltrate detected in the joints of RA patients. Initial analyses of cytokines released into the synovial membrane have shown an imbalance, with a predominance of pro-inflammatory mediators, indicating a deleterious effect of Th1 T cells (53). Beyond the well-studied B and T cells, immunological infiltration analysis reveals that patients with RA exhibit significantly elevated levels of various immune cells in synovial tissue compared to healthy individuals. These include CD56bright and CD56dim natural killer cells, central memory CD4 T cells, effector memory CD8 T cells, gamma delta T cells, macrophages, myeloid-derived suppressor cells (MDSCs), monocytes, natural killer cells, natural killer T cells, and plasmacytoid dendritic cells, all of which show statistically significant alterations (*p < 0.05*). Therefore, studying the immunological characteristics and changes in inflammatory cytokines in RA is essential for improving the diagnosis and treatment of the disease.

## 5 Conclusion

In this study, we employed bioinformatics and machine learning techniques to identify seven key genes associated with rheumatoid arthritis (RA): AKR1B10, MMP13, FABP4, NCF1, SPP1, COL1A1, and RASGRP1. These genes present promising potential as therapeutic targets for RA treatment. Our validation experiments using synovial tissues from collagen-induced arthritis (CIA) rats demonstrated that the traditional herbal medicine TYP has regulatory effects on these genes and mitigates histopathological changes in CIA rat tissues, underscoring its therapeutic efficacy against RA. Additionally, the analysis of TYP’s components reveals the complex mechanisms underlying traditional Chinese medicine, suggesting that further in-depth studies are needed to fully elucidate the molecular pathways through which TYP alleviates RA. This understanding could lead to the development of novel therapeutic strategies for RA treatment.

## Data Availability

The original contributions presented in the study are included in the article/Supplementary material, further inquiries can be directed to the corresponding authors.

## Footnotes

^1^ http://www.swissadme.ch/

^2^ https://www.ncbi.nlm.nih.gov/geo/

^3^ https://string-db.org/

^4^ https://www.rcsb.org/

^5^ https://pubchem.ncbi.nlm.nih.gov/

^6^ https://pga.mgh.harvard.edu/primerbank/

^7^ https://www.ncbi.nlm.nih.gov/

^8^ https://www.primer3plus.com/

^9^ https://blast.ncbi.nlm.nih.gov/
